# The 2019–2020 Australian forest fires are a harbinger of decreased prescribed burning effectiveness under rising extreme conditions

**DOI:** 10.1038/s41598-022-15262-y

**Published:** 2022-07-13

**Authors:** Hamish Clarke, Brett Cirulis, Trent Penman, Owen Price, Matthias M. Boer, Ross Bradstock

**Affiliations:** 1 Centre for Environmental Risk Management of Bushfires, Centre for Sustainable Ecosystem Solutions, University of Wollongong, Wollongong, NSW 2522 Australia; 2 NSW Bushfire Risk Management Research Hub, University of Wollongong, Wollongong, NSW 2522 Australia; 3 Hawkesbury Institute for the Environment, Western Sydney University, Locked Bag 1797, Penrith, NSW 2751 Australia; 4 FLARE Wildfire Research, School of Ecosystem and Forest Sciences, The University of Melbourne, Melbourne, Victoria 3363 Australia; 5 NSW Department of Planning and Environment, Science, Economics and Insights Division, Parramatta, NSW Australia

**Keywords:** Environmental sciences, Natural hazards

## Abstract

There is an imperative for fire agencies to quantify the potential for prescribed burning to mitigate risk to life, property and environmental values while facing changing climates. The 2019–2020 Black Summer fires in eastern Australia raised questions about the effectiveness of prescribed burning in mitigating risk under unprecedented fire conditions. We performed a simulation experiment to test the effects of different rates of prescribed burning treatment on risks posed by wildfire to life, property and infrastructure. In four forested case study landscapes, we found that the risks posed by wildfire were substantially higher under the fire weather conditions of the 2019–2020 season, compared to the full range of long-term historic weather conditions. For area burnt and house loss, the 2019–2020 conditions resulted in more than a doubling of residual risk across the four landscapes, regardless of treatment rate (mean increase of 230%, range 164–360%). Fire managers must prepare for a higher level of residual risk as climate change increases the likelihood of similar or even more dangerous fire seasons.

## Introduction

Intrinsic to the earth system for hundreds of millions of years, wildfires are increasingly interacting with humans and the things we value^1–3^. Mega-fires in recent years have caused loss of life and property and widespread environmental and economic impacts in many countries, challenging society’s ability to respond effectively^4–6^. Climate change has already caused changes in some fire regimes, with greater changes projected throughout this century^7–9^. There is a broad network of anthropogenic influences on fire likelihood, exposure and vulnerability including land-use planning, building construction and design, insurance, household and community actions, Indigenous cultural land management, ecosystem management, and research and development. Within this network, fire management agencies play a critical role in wildfire risk mitigation, although our understanding of the interactions between, and relative contributions of, these varied factors towards risk mitigation remains limited. Addressing these gaps is required to support the development and implementation of cost-effective risk management strategies^10^.

Prescribed burning is commonly used in contemporary fire management to alter fuels, with the intention of mitigating risks posed by wildfires to assets. This involves the controlled application of fire in order to modify fuel properties and increase the likelihood of suppressing any wildfires that subsequently occur in the area of the burn^11–13^. Although the effects and effectiveness of prescribed burning have come under intense scientific scrutiny^14^, major knowledge gaps remain in the design of locally tailored, cost-effective treatment strategies that aim to optimise risk mitigation across a range of management values^15^. Crucially, these values may sometimes be in conflict e.g. smoke health impacts from prescribed fire and wildfire^16^ or biodiversity conservation and asset protection^17^, necessitating methods for making trade-offs explicit^18^.

The 2019–2020 fires in south-eastern Australia resulted in 33 direct deaths, over 400 smoke-related premature deaths, the loss of over 3000 houses and new records for high severity fire extent and the proportion of area burnt for any forest biome globally^4,19–21^. These fires were an important opportunity to test the risk mitigation effects of prescribed burning. One empirical study found that about half the prescribed fires examined resulted in a significant decrease in fire severity, with effects greater for more recent burns and weaker for older burns^22^. Two other empirical studies^6,23^ found decreases in the probability of high severity fire and house loss after past fire (either prescribed fire or wildfire), but also that this effect was significantly weakened under extreme fire weather conditions, consistent with prior research^24,25^. Large ensemble fire behaviour modelling can complement these empirical studies by exploring far more variation in weather conditions, treatment strategies and ignition location than would be possible from the historical record^26–28^. Simulation modelling facilitates estimates of residual risk: the percentage of maximum bushfire risk remaining, in a given area, following a particular fire management scenario, with maximum typically based on a control scenario with no prescribed burning treatment^29^. Simulation modelling also enables tracking of the trajectory of risk in the aftermath of seasons such as the 2019–2020 one, where very large burned areas might be expected to have reduced landscape fuel loads and hence residual risk.

**Figure 1.**
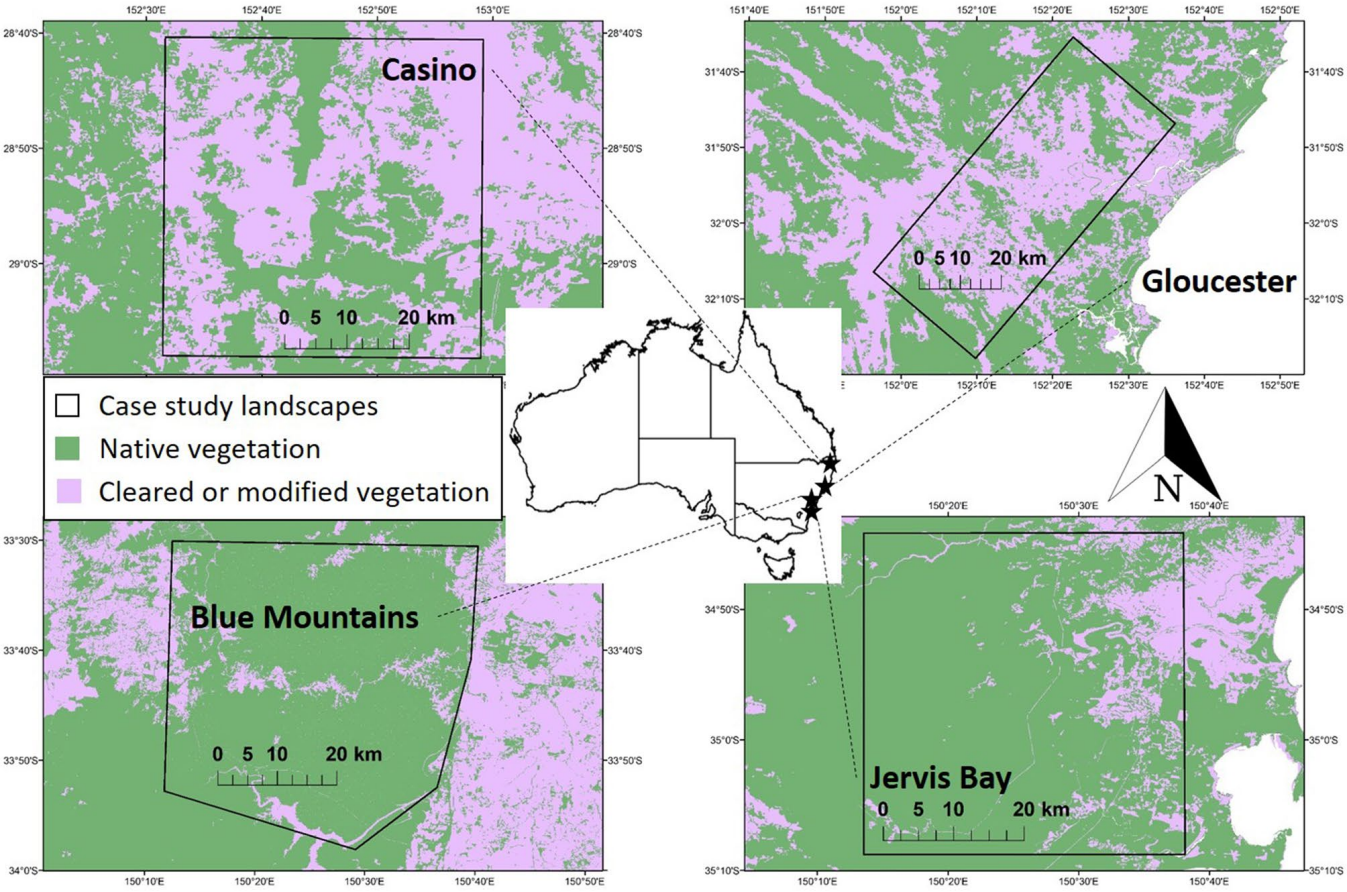
Fire behaviour simulations were carried out for four case study landscapes in south-eastern Australia: Casino, Gloucester, Blue Mountains and Jervis Bay. See Table 1 and Study Area in the Methods section for more information. This figure was generated using ArcGIS version 10.8 (https://www.esri.com/en-us/home).

Here we perform a simulation experiment on the effects of different rates of prescribed burning treatment on area burnt and the risks posed by wildfire to multiple values. We consider life, property and infrastructure across four case study landscapes (Fig. 1). In particular, we asked:

1. How much risk mitigation does prescribed burning provide in the weather conditions of 2019-20 compared to average fire season weather distributions, based on long-term records?
2. How much subsequent risk reduction did the Black Summer fires provide?
3. Over what time period will risk reduction be measurable?

## Results

### The effect of 2019-20 fire weather conditions on risk mitigation from prescribed burning

**Figure 2.**
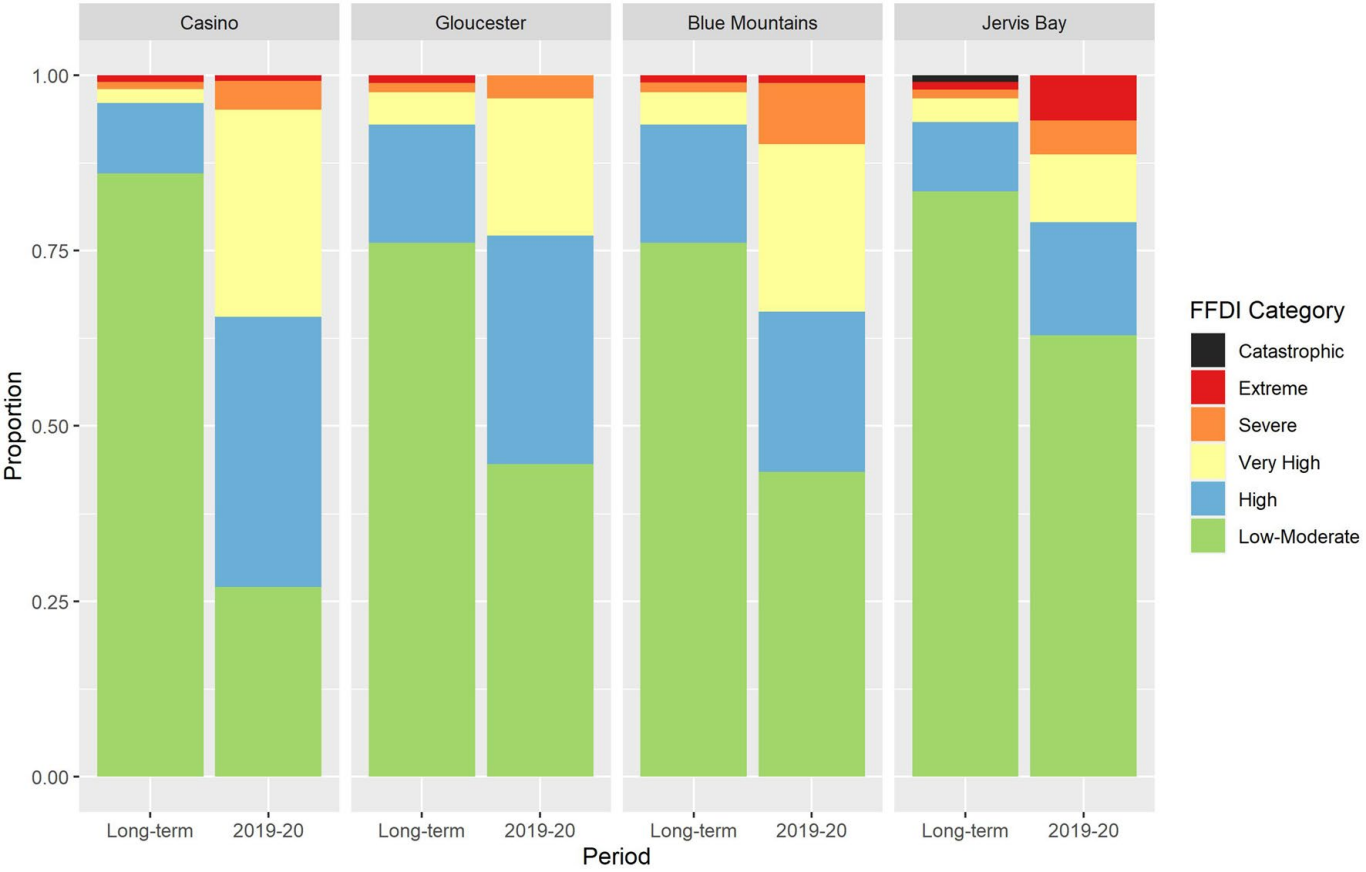
Relative frequency of FFDI categories from half-hourly weather station data during the long-term record (1995–2014 for Casino, 1991–2014 for Gloucester and Blue Mountains, 2000–2014 for Jervis Bay) and during the 2019–2020 season.

Fire weather conditions during the 2019–2020 season were markedly different to preceding years (Fig. 2). In all four case study landscapes there were fewer Low-Moderate days (Forest Fire Danger Index (FFDI): 0–12) and often considerably more High, Very High and Severe days (FFDI: 12–74). Only in the Jervis Bay landscape were there substantially more Extreme days (FFDI: 75–99) during the 2019–2020 season, while there were no Catastrophic days (FFDI ≥ 100) in any of the landscapes during 2019–2020.

**Figure 3.**
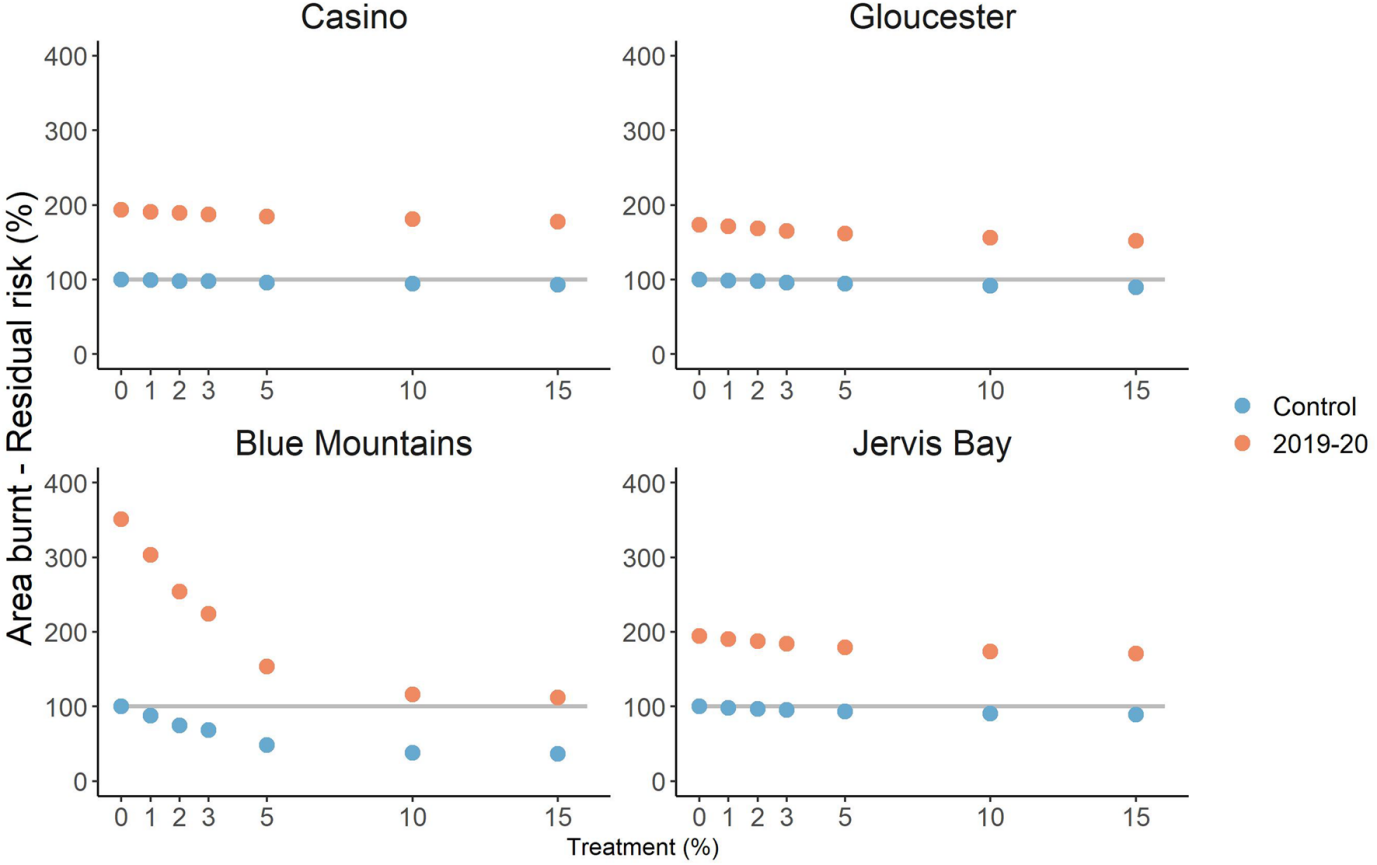
Residual risk trajectory of area burnt by wildfire in Casino, Gloucester, Blue Mountains and Jervis Bay. Risk is relative to a scenario with no prescribed burning and long-term weather (the 100% level on the y-axis). Markers represent different annual rates of treatment, colours represent different weather conditions (blue = control i.e. long-term, orange = 2019–2020 fire season).

**Figure 4.**
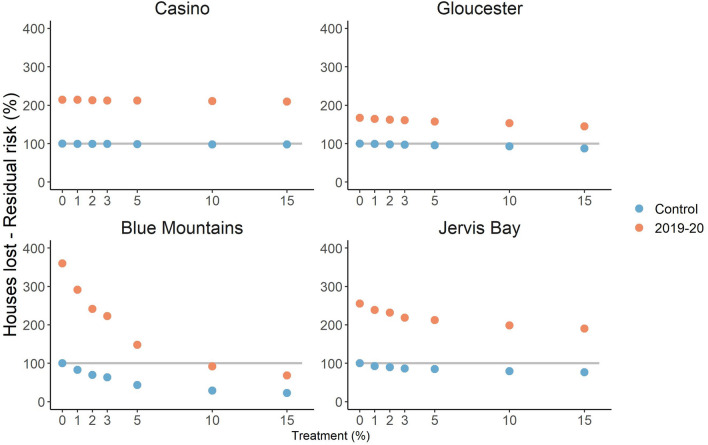
Residual risk trajectory of houses lost due to wildfire in Casino, Gloucester, Blue Mountains and Jervis Bay. Risk is relative to a scenario with no prescribed burning and long-term weather (the 100% level on the y-axis). Markers represent different annual rates of treatment, colours represent different weather conditions (blue = control i.e. long-term, orange = 2019–2020 fire season).

The 2019–2020 weather conditions strongly increased the residual risk of area burnt by wildfire and house loss due to wildfire (Figs. 3, 4). For any given treatment rate, the residual risk under 2019–2020 weather conditions far exceeded control conditions (i.e. conditions based on long-term historic weather). For area burnt there was a mean 220% increase in residual risk (range 170–351%), while for house loss the mean increase in residual risk was 244% (range 164–360%). Only under very high rates of treatment was prescribed burning under 2019–2020 conditions able to achieve a residual risk below that of zero treatment in the control scenario, and only for house loss in the Blue Mountains (Fig. 4). Elsewhere even the highest rates of treatment (well above rates achieved historically) resulted in a residual risk above that of zero treatment in the control scenario.

Prescribed burning resulted in a reduction in residual risk in all landscapes regardless of weather conditions, even though in almost all cases the risk remained higher than for zero treatment in the control scenario (see gradient of markers in Figs. 3, 4). The effect of increasing treatment was much stronger in the Blue Mountains, with a minimum residual risk of area burnt by wildfire under long-term weather conditions of 35%, and a minimum residual risk of house loss of 22%. In the other three landscapes the minimum residual risk was 89% for area burnt and 77% for house loss. The marginal effect of prescribed burning (i.e. the rate of change in risk mitigation with incremental changes in treatment rate) was greater under the extreme 2019–2020 weather conditions, even though the residual risk was much higher as described above. Results for life loss and infrastructure damage were similar (Supplementary Figures 1–3).

### Risk in the aftermath of 2019–2020 fire season

**Figure 5.**
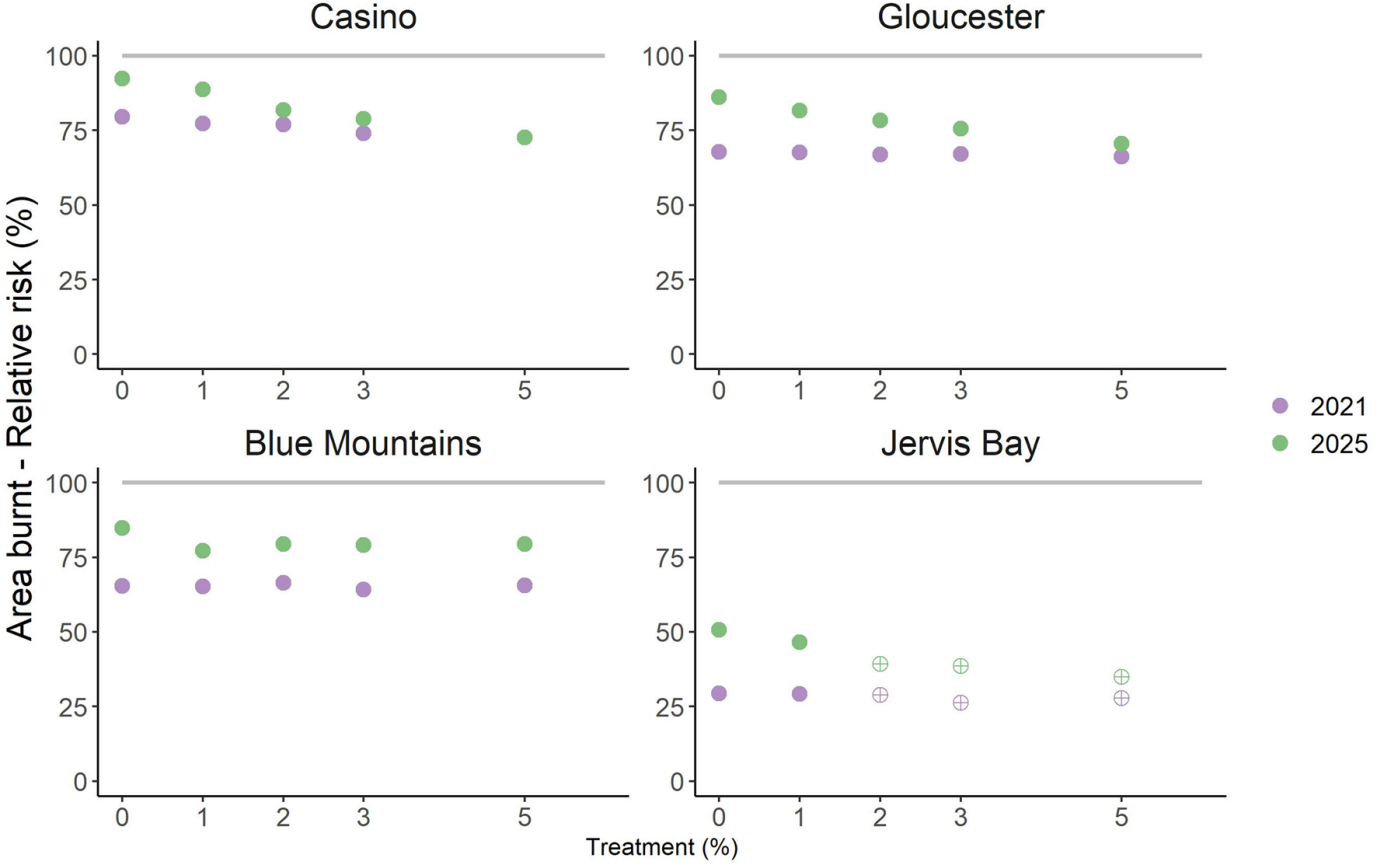
Future residual risk trajectory of area burnt by wildfire in the Casino, Gloucester, Blue Mountains and Jervis Bay case study areas. Risk is relative to a control scenario with pre-2019–2020 fuel load and no prescribed burning (the 100% level on the y-axis, indicated by line). Markers represent different annual treatment rates, colour indicates time period (blue = 2021 i.e. two years after 2019–2020 fire season, orange = 2025 i.e. six years after 2019–2020 fire season). In Jervis Bay the markers for 2, 3 and 5% p.a. treatment reflect edge treatment rates, with landscape treatment capped at 1% p.a. due to the very large area burnt during the 2019–2020 season (81% of the study area).

**Figure 6.**
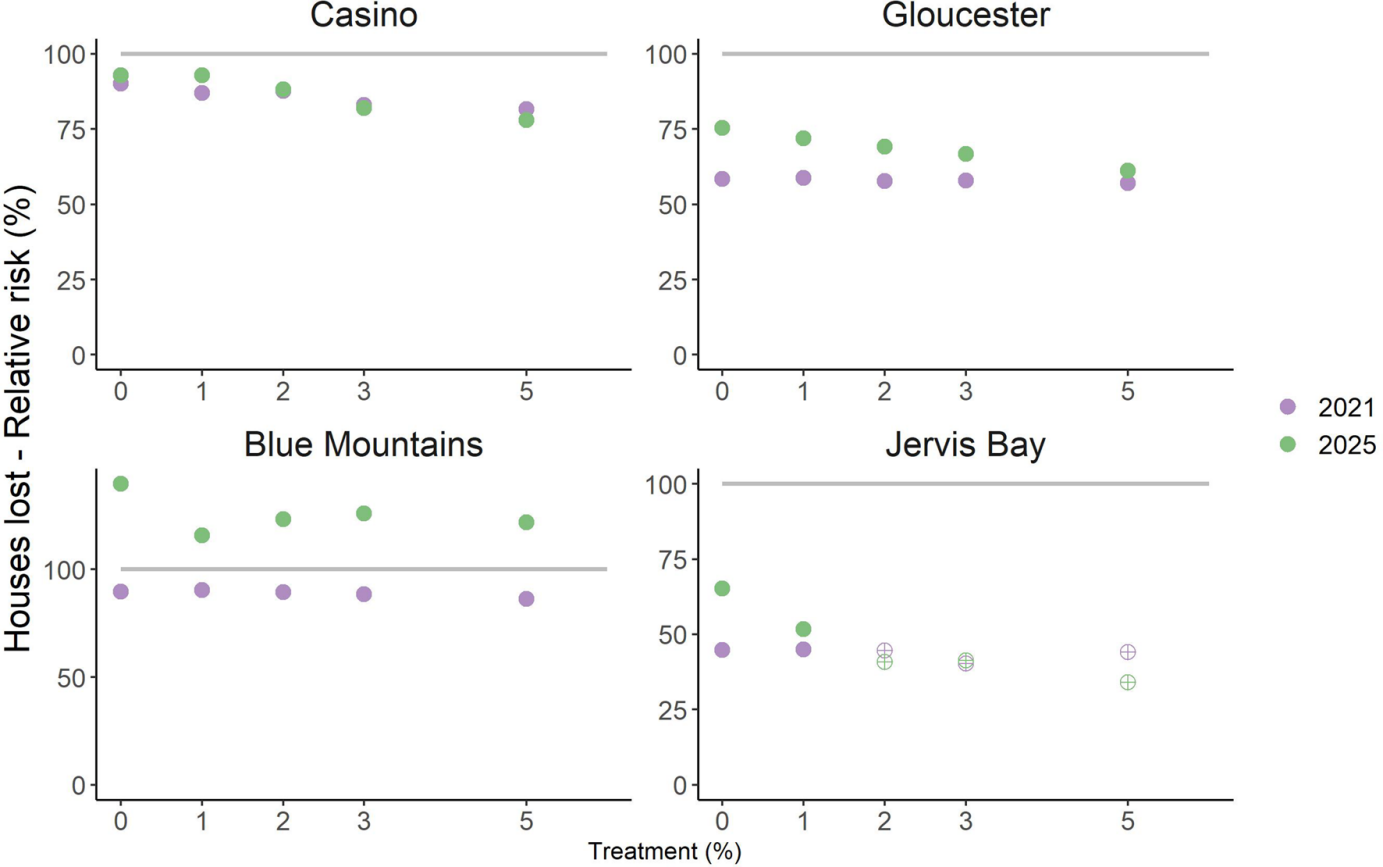
Future residual risk trajectory of houses lost due to wildfire in the Casino, Gloucester, Blue Mountains and Jervis Bay case study areas. Risk is relative to a control scenario with pre-2019–2020 fuel load and no prescribed burning (the 100% level on the y-axis, indicated by line). Markers represent different annual treatment rates, colour indicates time period (blue = 2021 i.e. two years after 2019–2020 fire season, orange = 2025 i.e. six years after 2019–2020 fire season). In Jervis Bay the markers for 2, 3 and 5% p.a. treatment reflect edge treatment rates, with landscape treatment capped at 1% p.a. due to the very large area burnt during the 2019–2020 season (81% of the study area).

The estimated fuel load reductions due to the 2019–2020 fire season were predicted to cause widespread short-term reductions in residual risk to area burnt by wildfire and house loss, regardless of treatment level (Figs. 5, 6). The potential area burnt by wildfire in 2021 was predicted to be at 30–80% of control (i.e. pre-2019–2020 levels) depending on landscape (Fig. 5, circles). The predicted reduction in area burnt was greatest in Jervis Bay and Gloucester, which experienced the greatest and second greatest proportion burnt during the 2019–2020 season respectively (Table 1). By 2025, the residual risk of area burnt by wildfire climbed to 50–90% of control levels across the four study areas (Fig. 6). Results are similar for house loss (Fig. 6) i.e. the reductions in future wildfire risk due to the 2019–2020 season are partial and temporary, with residual risk actually exceeding control levels in the Blue Mountains by 2025. The re-accumulation of fuel over time is predicted to lead to greater risk mitigation from prescribed burning by 2025 than by 2021 (compare the gradients of the crosses and the circles in Figs. 5, 6). As with the previous analysis, results for life loss and infrastructure damage were similar (Supplementary Figures 4-6).

**Table 1:** Selected case study properties.

Case study landscape	Area (ha) (% burnt in 2019–2020)	Elevation (m.a.s.l.)	Mean annual rainfall (mm)	Mean annual T_max_ (℃)	Mean annual T_min_ (℃)
Casino	228,000 (30%)	159 (16–662)	1055 (983–1250)	24.4 (21.1–25.5)	12.4 (11.3–13.3)
Gloucester	209,000 (63%)	120 (11–844)	1147 (1018–1672)	22.6 (18.5–24.2)	11.6 (8.8–12.8)
Blue Mountains	191,000 (63%)	531 (2–1131)	1023 (738–1271)	19.6 (16.0–23.1)	8.4 (6.9–10.3)
Jervis Bay	170,000 (81%)	186 (− 10 to 788)	1021 (876–1348)	20.3 (17.3–22.0)	9.9 (6.5–12.8)

For mean annual maximum and minimum temperature, area average and range are shown.

## Discussion

Weather conditions during the 2019–2020 Australian fire season were a substantial risk multiplier compared to long-term weather conditions. The relative risks due to wildfire, quantified in terms of area burnt or house loss, doubled in three of four forested landscapes and more than tripled in the other. While prescribed burning partially mitigated these risks, the effect size was typically dwarfed by the effect of extreme weather conditions. In most cases zero treatment under long-term historic weather conditions yielded a lower residual risk than even the highest prescribed burning rates when combined with the 2019–2020 fire weather conditions. We also found that wildfire risk was likely to be reduced in the aftermath of the 2019–2020 fires, based on the implied fuel reduction associated with the unprecedented area burnt during the 2019–2020 season. However, the residual risk was still substantial in some areas and was predicted to rise steadily in the coming years, regardless of prescribed burning treatment rates.

Prescribed burning can mitigate a range of risks posed by wildfire, however residual risk can be substantial and is likely to increase strongly during severe fire weather conditions^6,24^. We found that the risk mitigation available from prescribed burning varies considerably depending on where it is carried out and which management values are being targeted, consistent with previous modelling studies that suggest there is no ‘one size fits all’ solution to prescribed burning treatment^15,16^. Of the factors influencing regional variation in prescribed burning effectiveness, the configuration of assets and the type, amount and condition of native vegetation are likely to be important. The Blue Mountains landscape, where area burnt by wildfire responded most strongly to treatment, has a relatively high proportion of native vegetation compared to the other landscapes, particularly Casino and Gloucester which are mostly cleared. The Blue Mountains also has an unusual combination of a high population concentrated in a linear strip of settlements surrounded by forest, which may contribute to greater returns on treatment (Fig. 1). Future research could systematically investigate the relationship between risk mitigation and properties of key variables such as asset distribution, vegetation and burn blocks for an expanded selection of landscapes. Although residual risk was greatly reduced in some areas after the 2019–2020 fire season, it remained substantial in other areas and was generally predicted to rise rapidly with fuel re-accumulation over the following five years. More work is needed to understand potential feedbacks between increasing fire activity, fuel accumulation and subsequent fire activity^8^.

Our conclusions are dependent on a number of assumptions associated with our fire behaviour simulation approach, including the foundational premise that fire spread is a function of fire weather, fuel load and factors such as topography. Fire behaviour simulators built on these assumptions have known biases and perform better when these are addressed, although their tendency to underestimate extreme fire behaviour suggests our results may be conservative^30–32^. The approach also assumes that both wildfires and prescribed burns consume equivalent quantities of fuel and that this fuel starts to re-accumulate after fire as a negative exponential function of time since fire, eventually stabilising at an equilibrium amount. In fact fuel consumption rates vary considerably within a given fire but also between wildfires and prescribed fires, which consume less fuel^33,34^. This also points towards our results being conservative due to potentially overestimating the mitigation effect of prescribed burning. Furthermore the accumulation of fuel post fire depends on the vegetation type, soil and climate^35^. Our experiments on the trajectory of risk after the 2019–2020 fire season may be limited by the relatively short amount of time allowed to elapse, which may be insufficient for prescribed burning treatment effects to become apparent. More broadly, our study design involves repeated instances of a single wildfire and thus does not capture the fire regime i.e. the effects of multiple fires in space and time, nor does it factor in future changes in climate, fuel or fuel moisture^36^. We did not model suppression, which is a complex function of fuel type, fuel load, fire behaviour, weather, topography and fire management decision making^37^. Suppression can reduce a range of risks although it is less effective under extreme weather conditions^38–40^.

Fire-prone landscapes around the world have experienced increasingly severe fire weather conditions^20,41^. The extreme conditions of the 2019–2020 fire season are projected to occur more frequently in the 21st century^42^. Our results suggest that climate change could seriously undermine the role played by prescribed burning in wildfire risk mitigation, as found in previous studies^43,44^. Using landscape simulation modelling in the Blue Mountains and the Woronora Plateau (about 100 km north of our Jervis Bay landscape), Bradstock et al.^43^ found that the rate of prescribed burning treatment would need to quintuple or more by 2050 to counteract the effects of climate change on risk mitigation in terms of measures such as area burned and intensity of unplanned fire. Our study assumes that similar or greater treatment rates will be possible in future, which may not be the case depending on the prevalence of suitable prescribed burning weather conditions^45,46^. These findings demonstrate that there can be no wildfire risk mitigation without effective climate change mitigation^47^. Our research reinforces the need for comprehensive, transparent and objective evaluation of the effectiveness of existing attempts to mitigate wildfire risk across a range of management objectives, with future work potentially targeting additional management values such as smoke production and associated health impacts, agriculture and tourism impacts, and more nuanced measures of environmental impact. Such an evaluation could inform the trial and implementation of a range of locally tailored risk mitigation measures that address the full complexity of fire across preparation, response and recovery phases, such as prescribed burning, mechanical fuel reduction, anthropogenic ignition management, suppression, planning, construction and community engagement.

## Methods

### Study area

We selected four case study landscapes that were extensively impacted during the 2019–2020 fire season: Casino (69,362 ha burnt), Gloucester (132, 281 ha), Blue Mountains (119,626 ha) and Jervis Bay (137,049 ha) (Fig. 1; Table 1). All landscapes are forested, have considerable Wildland Urban Interface (WUI), and have a history of both wildfire and prescribed fire. Case study landscapes were approximately 200,000 ha (Table 1), intended to align with the upper limit of the size distribution of wildfires in local ecosystems (During the 2019–2020 fire season the Gospers Mountain fire, the result of mergers between several large fires in the Blue Mountains World Heritage Area and neighbouring areas, had a final burned area of over 500,000 ha).

The dominant land cover in the Casino landscape is cleared or modified vegetation (58%). The main native vegetation is dry sclerophyll forest with a shrub/grass understorey (17% of the study area) followed by wet sclerophyll forest with a grassy understorey (9%). The Casino area has a population of about 12,000, mostly concentrated in the town of Casino with a small number dispersed on semi-rural properties. Cleared or modified vegetation is also the dominant land cover in the Gloucester landscape (60%). The main native vegetation is wet sclerophyll forest with a grassy understorey (23% of the study area) followed by wet sclerophyll forest with a shrubby understorey (8%). The population is about 30,000, most of which live in the town of Taree on the eastern edge of the landscape with the remainder in smaller towns and semi-rural properties. The main native vegetation in the Blue Mountains landscape is dry sclerophyll forest with a shrubby understorey (63% of the study area) followed by dry sclerophyll forest with a shrub/grass understorey (9%). About 11% of the landscape is cleared or modified vegetation. Around 100,000 people live within the area, mainly living in a string of suburbs along a highway which bisects the region. The main native vegetation in the Jervis Bay landscape is dry sclerophyll forest with a shrubby understorey (40% of the study area) followed by wet sclerophyll forest with a grassy understorey (17%). Around 14% of the landscape is cleared or modified vegetation. About 50,000 people live within the area, mostly in the township of Nowra in the northeast with most of the remainder in coastal suburbs in the southeast.

All four landscapes are examples of the temperature eucalypt forest fire regime niche, characterised by high-productivity, with infrequent low-intensity litter fires in spring and medium-intensity shrub fires in spring and summer^48^. Fire intensity typically ranges from 1000 to 5000 kW m^−1^, although extreme weather conditions may support crown fires where fire intensity can reach 10,000–50,000 kW m^−1^. Fire interval is around 5–20 years, although can be as long as 20–100 years^48^. Contemporary prescribed burning rates average 2.5% p.a. in the Blue Mountains landscape and range from 0.4 to 0.6% p.a.in the Casino, Gloucester and Jervis Bay landscapes.

### Phoenix fire simulator

Fires were simulated using PHOENIX RapidFire v4.0.0.7^49^, which is commonly applied in operations across south-eastern Australian states, including NSW^17^. Fire growth and rate of spread are calculated from Huygens’ propagation principle of fire edge^50^, a modified McArthur Mk5 forest fire behaviour model^51,52^ and a generalisation of the CSIRO southern grassland fire spread model^53^. A 30-m resolution digital elevation model was included to allow PHOENIX to incorporate topographic effects on fire behaviour. Vegetation mapping and fuel accumulation models for major vegetation types of the case study landscapes were supplied by the NSW Rural Fire Service. Simulations were run at 180m grid resolution and model output included flame length, ember density, convection and intensity.

### Scenario parameterisation

PHOENIX estimates fuel loads using separate fuel accumulation curves for combined surface and/near-surface, elevated and bark fuels^54^. These curves are based on a negative exponential growth function and varied among vegetation types^55^. The treatable portion of each case study landscape was defined as all fuels except crop, farm and urban landcover, and comprised 38% of the Casino landscape, 52% of the Gloucester landscape, 70% of the Blue Mountains landscape and 83% of the Jervis Bay landscape. Treatable fuels were separated into two types of management-sized ‘burn blocks’. Edge blocks were adjacent to property and settlements, while landscape blocks were more remote and larger. For edge blocks, a minimum burn interval of 5 years was used as it reflects what is feasible for agencies to achieve while allowing fuel recovery after burning. For landscape blocks, the minimum burn interval is the minimum tolerable fire interval for the majority of the vegetation type within each block, as represented by NSW Department of Planning and Environment mapping. In each case study landscape, 1000 ignition locations were selected based on an empirical model developed and tested for similar forest types^56^. Individual fires were ignited at 11:00 h local time and propagated for 12 h, unless self-extinguished within this period. This time period provides a standardised approach for risk estimation^15,57^ and was chosen as a compromise between a sufficient amount of time for significant wildfire impacts to be realised^58^, while avoiding the factorial multiplication of weather conditions spanning multiple days. We tested seven combinations of equal edge and landscape treatment (0, 1, 2, 3, 5, 10, 15% p.a.), resulting in a range of fuel age classes for each simulation (Supplementary Figures 7–10). Half-hourly weather data was drawn from the full record of observations at the nearest Bureau of Meteorology automatic weather station for each case study landscape (Casino 1995–2014, Gloucester 1991–2014, Blue Mountains 1991–2014, Jervis Bay 2000–2014). Simulations were repeated for each of the fire danger categories that had been recorded during the fire season (Spring-Summer) in each case study landscape i.e. Low–Moderate (0–11), High (12–24), Very High (25–49), Severe (50–74), Extreme (75–99) and in Jervis Bay only, Catastrophic (100+). The results from the simulated fires were used to estimate the impact on five management values (see “Impact estimation” section below) and then adjusted for the frequency of fire weather conditions contributing to ignitions and fire spread to estimate annualised risk (see “Risk estimation” section below).

Two sets of simulations were run to explore the effect of 2019–2020 fire weather conditions on prescribed burning effectiveness: (1) with weather drawn from the long-term historical record of fire season observations, referred to as "control”, (2) with weather drawn only from the 2019–2020 fire season, referred to as “2019–2020”. For Casino the period of active fire in the 2019–2020 fire season was September 2019 to December 2019, for Gloucester and the Blue Mountains this was October 2019 to December 2019, and for Jervis Bay this was December 2019 to January 2020. The relative frequency of fire weather conditions in each scenario was incorporated into risk estimation through a Bayesian decision network (see “Risk estimation” section below).

Three sets of simulations were run to explore the trajectory of risk in the aftermath of 2019–2020 fire season: (1) with a fire history excluding the 2019–2020 fire season and with no prescribed burning, referred to as “control”, (2) with a fire history including the 2019–2020 fire season, and with prescribed burning and fuel accumulation through to 2021 i.e. 2 years after the 2019–2020 season (“2021”), and (3) the same as (2) except through to 2025 (“2025”). Due to the very large area burnt during the 2019–2020 season, prescribed burning treatment rates (edge and landscape) were capped at 5% p.a. for Casino, Gloucester and the Blue Mountains. In Jervis Bay, where 81% of the study area was burned by the 2019–2020 fires, edge treatment was capped at 5% p.a. and landscape treatment rate was capped at 1% p.a.

### Impact estimation

Effectiveness of prescribed burning at mitigating wildfire impacts was assessed base on area burnt and four management values: house loss, loss of human life, length of powerline damaged and length of road damaged. Area burnt was a direct output from the fire behaviour simulations. The probability of house loss was calculated as a function of predicted ember density, flame length and convection as presented in^59^. House loss was calculated per 180-m model grid cell and then multiplied by the number of houses in that grid cell to estimate the number of houses lost per fire. Statistical loss of human life was based on house loss (using the house loss function), the number of houses exposed (using simulation output) and the number of people exposed to fire^60^. House location and population density data were derived from national datasets (^61^, Australian Bureau of Statistics) and combined to give the total number of people exposed to fire. Road and powerline location data was supplied by the NSW Department of Planning and Environment. In the absence of empirical data a simple threshold of 10,000 kW/m was used to classify roads or powerlines within each 180-m grid cell as damaged by fire or not. Impacts were estimated from simulation output and the datasets described above, resulting in a distribution of area burnt and impacts on the four management values, corresponding to different weather, treatment and ignition scenarios.

### Risk estimation

Building on previous studies^15,57^, a Bayesian Decision Networks (BDN) approach was used to generate residual risk estimates and hence evaluate the risk mitigation available from prescribed burning. We adopted the recommendations of Marcot et al.^62^ and Chen and Pollino^63^ in designing our BDN. A conceptual model was adapted from previous studies of fire management^64^ and used to create an influence diagram. In this model fire weather affected ignition probability; fire weather and treatment option (a decision node) affected the distribution of fire sizes; and fire weather, fire size and fire management affected the amount of loss for a given management value. To translate the influence diagram into risk estimates, probability distribution tables were populated for the fire weather node (based on weather station data) and the fire size and management value impact nodes (based on the impact estimation step described above) of the BDN. The BDN then generated output values for each of the different prescribed burning treatment scenarios, based on the influence diagram.

Continuous data were discretised on a log scale across the range of values iteratively to get a relatively even distribution across non-zero values. For each FFDI category, we calculated the average maximum daily FFDI during the fire season for each study area, using the same weather station data used to drive PHOENIX. FFDI values were then separated into fire days (fire recorded within 200 km of the weather station) and non-fire days. The relative frequency of fire days was then used to drive ignitions in the BDN. Raw risk values were the expected node likelihoods for area burnt, house loss, life loss, length of powerline damaged and length of road damaged. These raw values were converted into residual risk values by dividing them by the risk value associated with the zero edge, zero landscape treatment scenario. These risks can be validly compared between regions because they reflect the observed distribution of fire weather conditions in each area. Further details of fire behaviour simulations, impact estimation and risk estimation can be found in^15,57^.

## Supplementary Information

Supplementary Information.

## Data Availability

The datasets generated from fire simulation and risk estimation for the current study are available from the corresponding author on reasonable request. Weather data is available from the Australian Bureau of Meteorology (http://www.bom.gov.au). Vegetation mapping and fuel accumulation models are available from the NSW Rural Fire Service (https://www.rfs.nsw.gov.au). Fire-sensitive vegetation, road and powerline location data is available from the NSW Department of Planning and Environment (https://www.environment.nsw.gov.au).

## Code availability

Code to prepare the plots is available on request.
