# Supplementary material for: The 2019–2020 Australian forest fires are a harbinger of decreased prescribed burning effectiveness under rising extreme conditions
Source: Sci Rep. 2022 Jul 13;12:11871. doi: 10.1038/s41598-022-15262-y (PMC9279303; doi:10.1038/s41598-022-15262-y)
Supplement: Supplementary file 1 — Supplementary Information. [file 41598_2022_15262_MOESM1_ESM.pdf]

The 2019-20 Australian forest fires are a harbinger of decreased prescribed burning effectiveness under rising extreme conditions

Hamish Clarke, Brett Cirulis, Trent Penman, Owen Price, Matthias Boer, Ross Bradstock

Supplementary Material

Supplementary Figure 1 Residual risk trajectory of lives lost due to wildfire

Supplementary Figure 2 Residual risk trajectory of powerline damage due to wildfire

Supplementary Figure 3 Residual risk trajectory of road damage due to wildfire

Supplementary Figure 4 Future residual risk trajectory of lives lost due to wildfire

Supplementary Figure 5 Future residual risk trajectory of powerline damage due to wildfire

Supplementary Figure 6 Future residual risk trajectory of road damage due to wildfire

Supplementary Figure 7 Representative fire histories in Casino

Supplementary Figure 8 Representative fire histories in Gloucester

Supplementary Figure 9 Representative fire histories in the Blue Mountains

Supplementary Figure 10 Representative fire histories in Jervis Bay

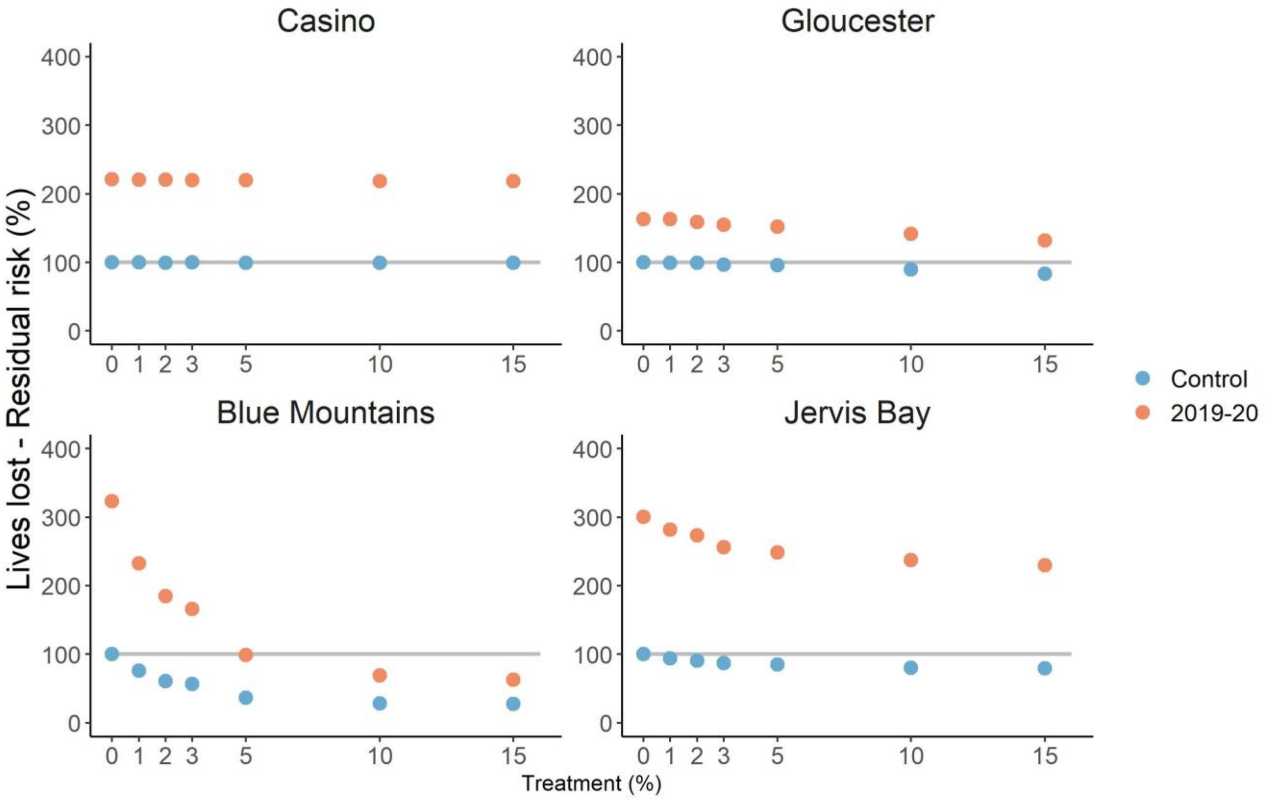

Supplementary Figure 1. Residual risk trajectory of lives lost due to wildfire in Casino, Gloucester, Blue Mountains and Jervis Bay. Risk is relative to a scenario with no prescribed burning and long-term weather (the 100% level on the y-axis). Markers represent different annual rates of treatment, colours represent different weather conditions (blue = control i.e. long-term, orange = 2019-20 fire season).

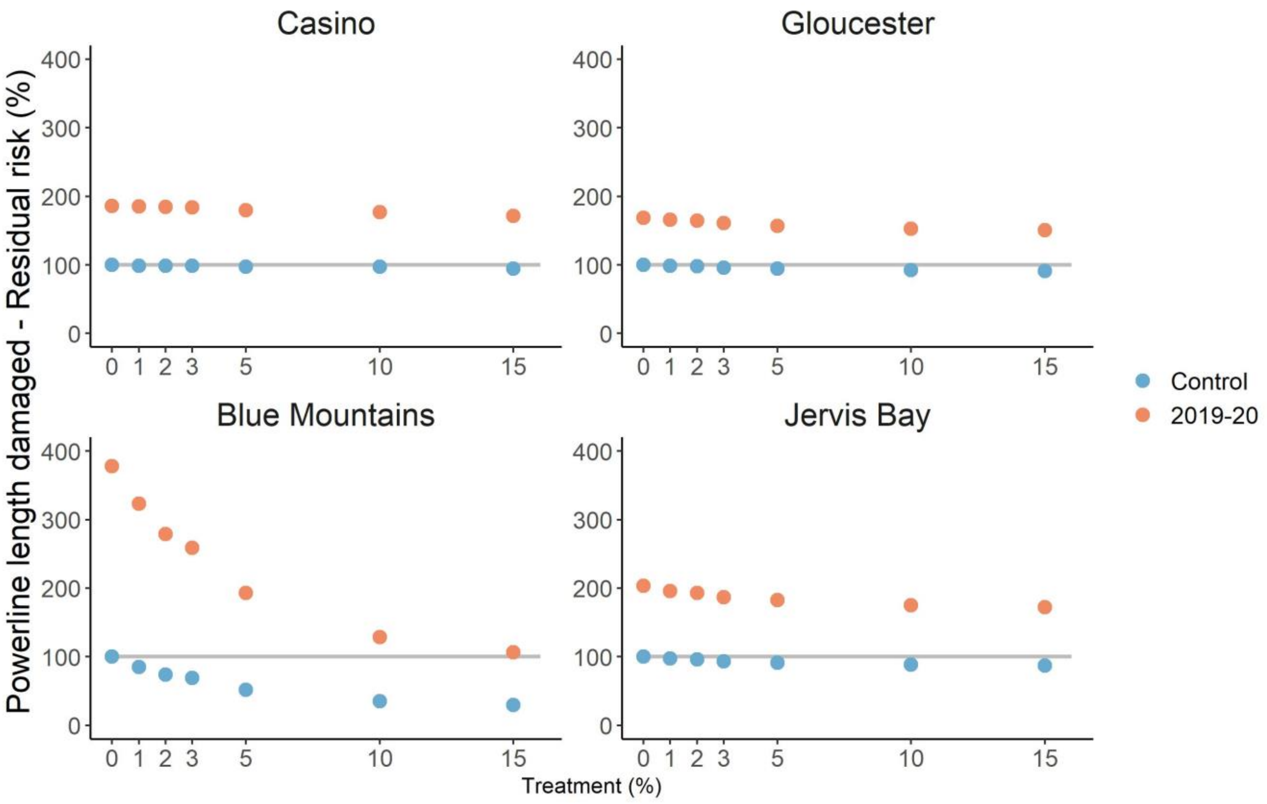

Supplementary Figure 2. Residual risk trajectory of powerline damage due to wildfire in Casino, Gloucester, Blue Mountains and Jervis Bay. Risk is relative to a scenario with no prescribed burning and long-term weather (the 100% level on the y-axis). Markers represent different annual rates of treatment, colours represent different weather conditions (blue = control i.e. long-term, orange = 2019-20 fire season).

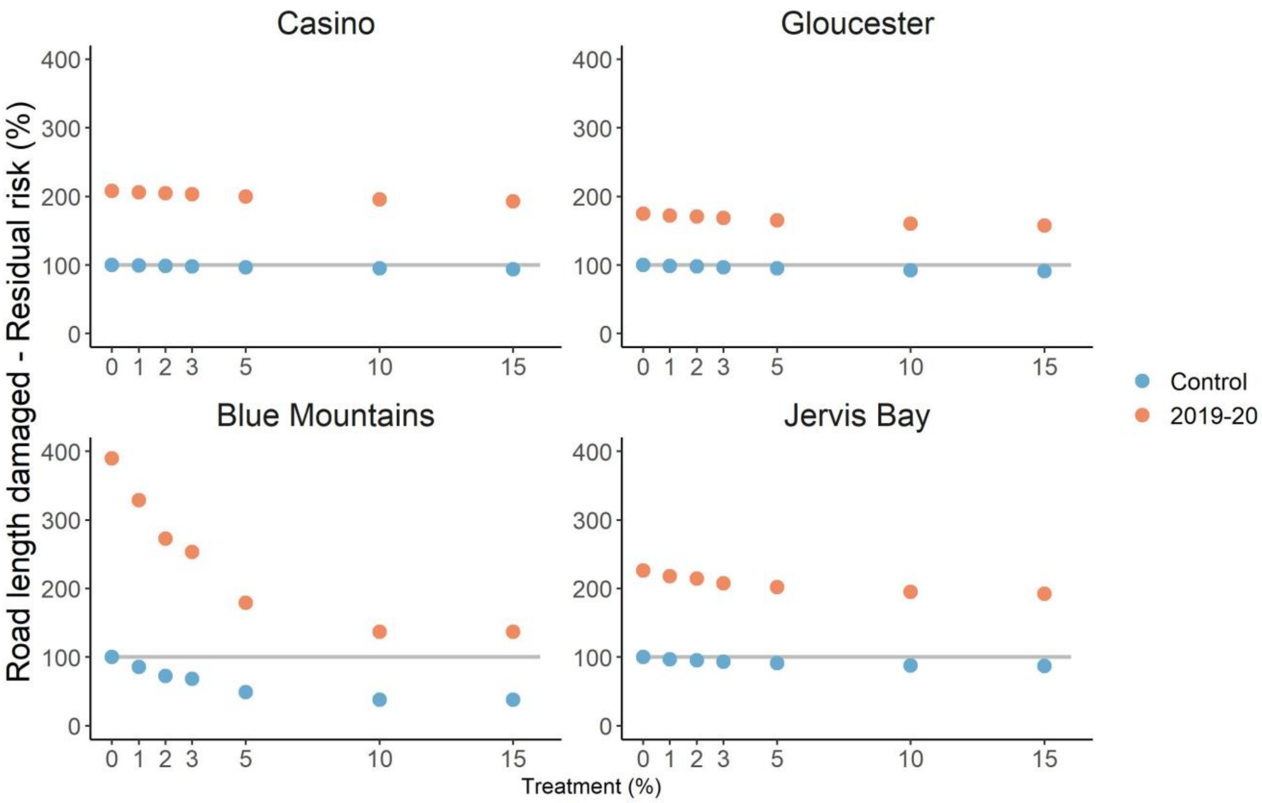

Supplementary Figure 3. Residual risk trajectory of road damage due to wildfire in Casino, Gloucester, Blue Mountains and Jervis Bay. Risk is relative to a scenario with no prescribed burning and long-term weather (the 100% level on the y-axis). Markers represent different annual rates of treatment, colours represent different weather conditions (blue = control i.e. long-term, orange = 2019-20 fire season).

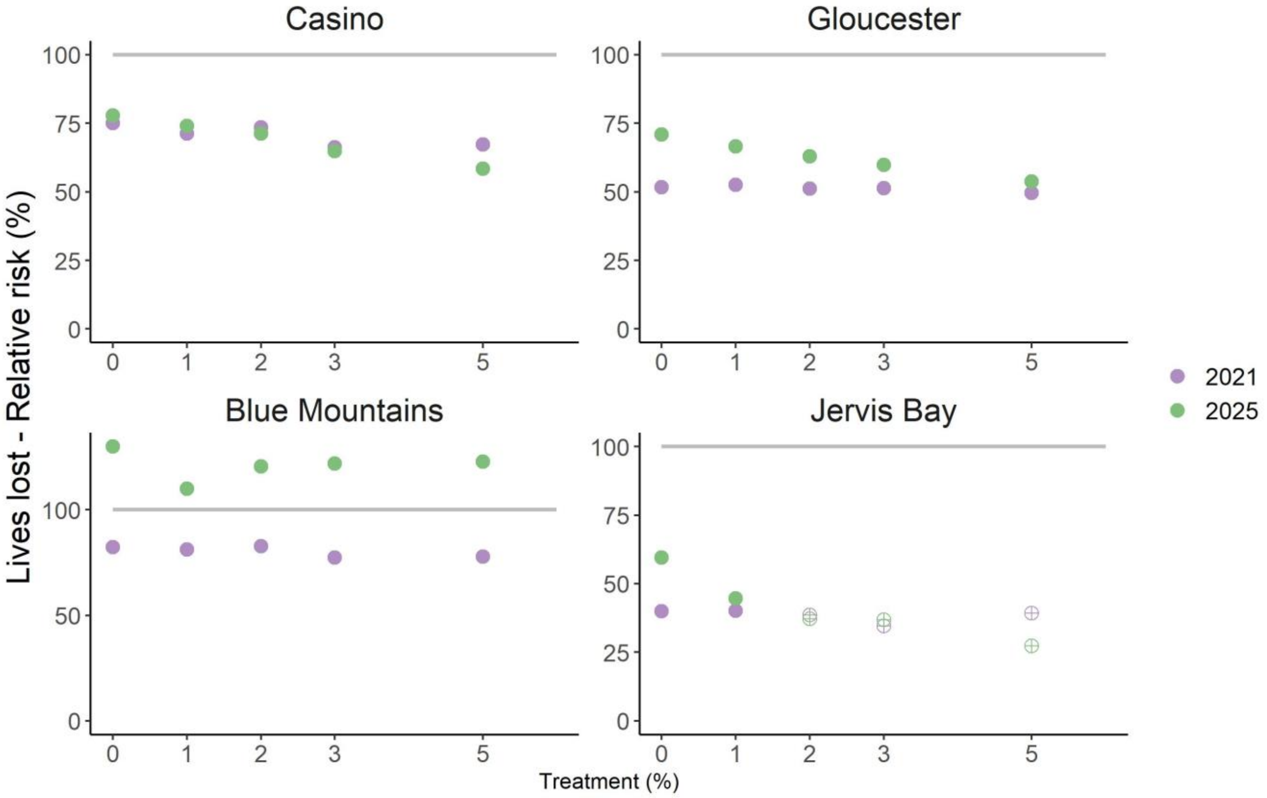

Supplementary Figure 4. Future residual risk trajectory of lives lost due to wildfire in the Casino, Gloucester, Blue Mountains and Jervis Bay case study areas. Risk is relative to a control scenario with pre-2019-20 fuel load and no prescribed burning (the 100% level on the y-axis, indicated by line). Markers represent different annual treatment rates, colour indicates time period (blue = 2021 i.e. two years after 2019-20 fire season, orange = 2025 i.e. six years after 2019-20 fire season). In Jervis Bay the markers for 2, 3 and 5% p.a. treatment reflect edge treatment rates, with landscape treatment capped at 1% p.a. due to the very large area burnt during the 2019-20 season (81% of the study area).

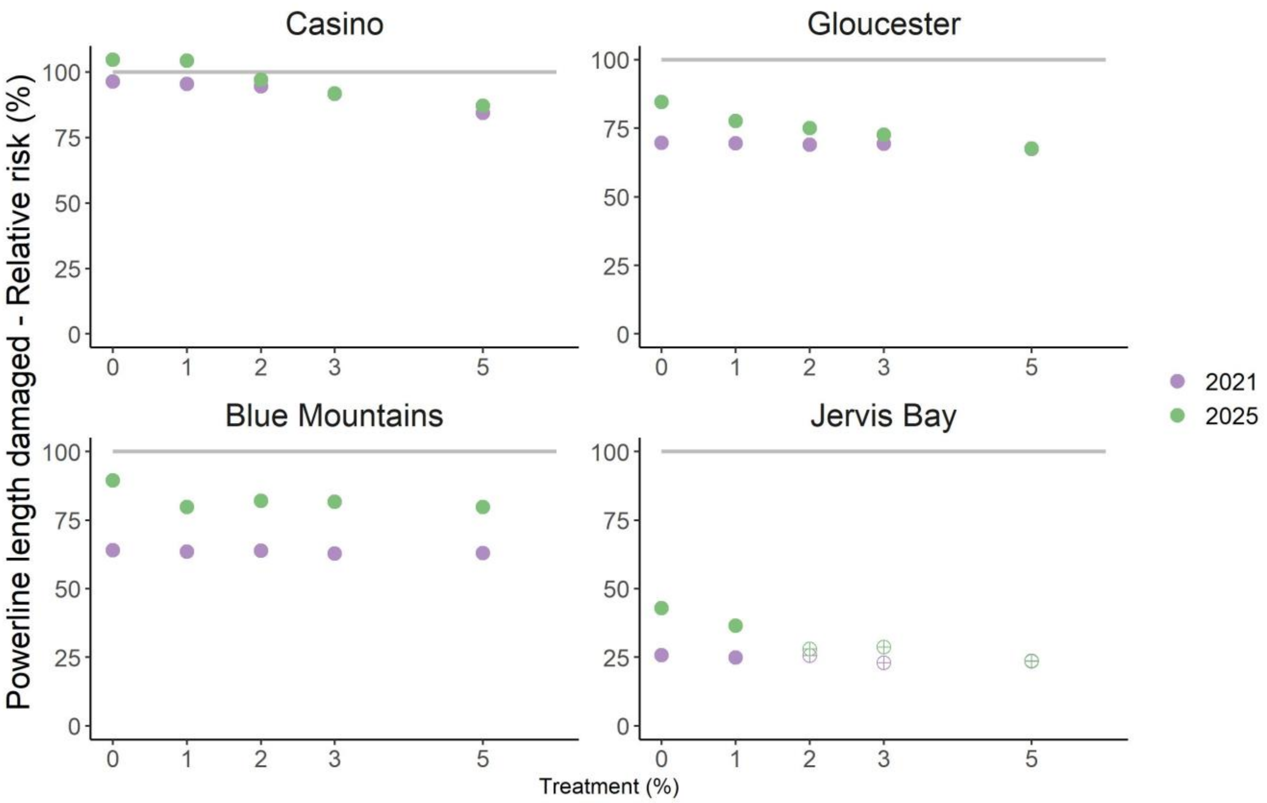

Supplementary Figure 5. Future residual risk trajectory of powerline damage due to wildfire in the Casino, Gloucester, Blue Mountains and Jervis Bay case study areas. Risk is relative to a control scenario with pre-2019-20 fuel load and no prescribed burning (the 100% level on the y-axis, indicated by line). Markers represent different annual treatment rates, colour indicates time period (blue = 2021 i.e. two years after 2019-20 fire season, orange = 2025 i.e. six years after 2019-20 fire season). In Jervis Bay the markers for 2, 3 and 5% p.a. treatment reflect edge treatment rates, with landscape treatment capped at 1% p.a. due to the very large area burnt during the 2019-20 season (81% of the study area).

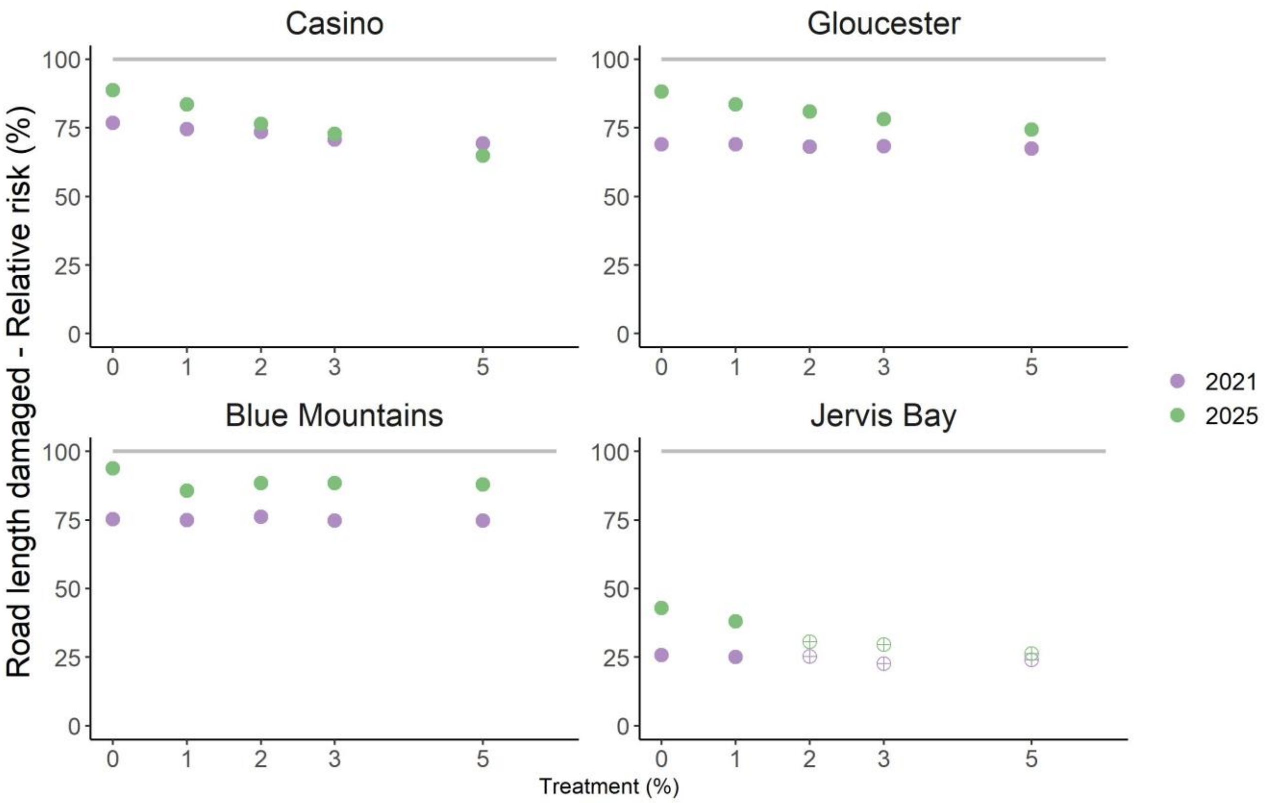

Supplementary Figure 6. Future residual risk trajectory of road damage due to wildfire in the Casino, Gloucester, Blue Mountains and Jervis Bay case study areas. Risk is relative to a control scenario with pre-2019-20 fuel load and no prescribed burning (the 100% level on the y-axis, indicated by line). Markers represent different annual treatment rates, colour indicates time period (blue = 2021 i.e. two years after 2019-20 fire season, orange = 2025 i.e. six years after 2019-20 fire season). In Jervis Bay the markers for 2, 3 and 5% p.a. treatment reflect edge treatment rates, with landscape treatment capped at 1% p.a. due to the very large area burnt during the 2019-20 season (81% of the study area).

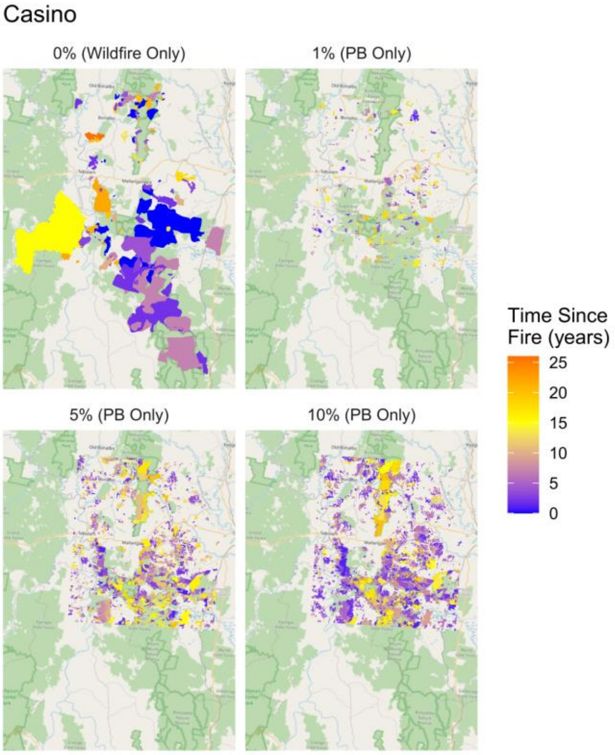

Supplementary Figure 7. Representative fire histories in Casino for the analysis of the effects of 2019-20 weather conditions on prescribed burning effectiveness. All simulations include wildfire history (top left). The fire history and fuel age class distribution is shown for three prescribed burning treatment strategies: 1% edge and 1% landscape (top right), 5% edge and 5% landscape (bottom left) and 10% edge and 10% landscape (bottom right). This figure was generated using ArcGIS version 10.8 (https://www.esri.com/en-us/home).

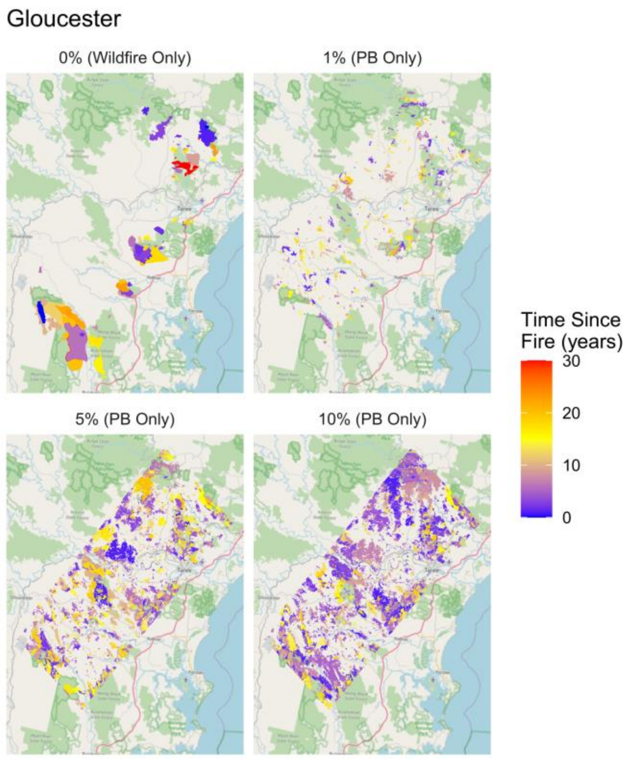

Supplementary Figure 8. Representative fire histories in Gloucester for the analysis of the effects of 2019-20 weather conditions on prescribed burning effectiveness. All simulations include wildfire history (top left). The fire history and fuel age class distribution is shown for three prescribed burning treatment strategies: 1% edge and 1% landscape (top right), 5% edge and 5% landscape (bottom left) and 10% edge and 10% landscape (bottom right). This figure was generated using ArcGIS version 10.8 (https://www.esri.com/en-us/home).

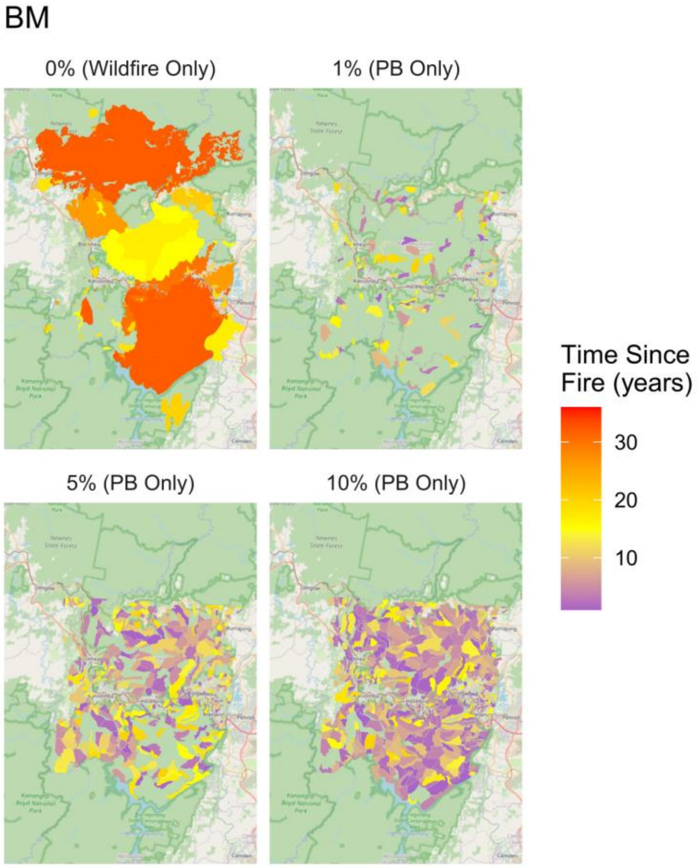

Supplementary Figure 9. Representative fire histories in the Blue Mountains for the analysis of the effects of 2019-20 weather conditions on prescribed burning effectiveness. All simulations include wildfire history (top left). The fire history and fuel age class distribution is shown for three prescribed burning treatment strategies: 1% edge and 1% landscape (top right), 5% edge and 5% landscape (bottom left) and 10% edge and 10% landscape (bottom right). This figure was generated using ArcGIS version 10.8 (https://www.esri.com/en-us/home).

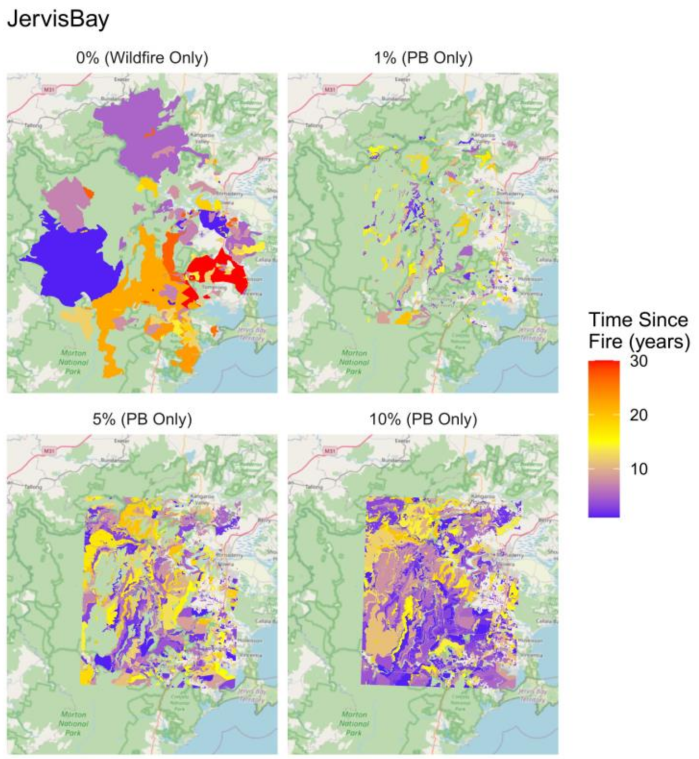

Supplementary Figure 10. Representative fire histories in Jervis Bay for the analysis of the effects of 2019-20 weather conditions on prescribed burning effectiveness. All simulations include wildfire history (top left). The fire history and fuel age class distribution is shown for three prescribed burning treatment strategies: 1% edge and 1% landscape (top right), 5% edge and 5% landscape (bottom left) and 10% edge and 10% landscape (bottom right). This figure was generated using ArcGIS version 10.8 (https://www.esri.com/en-us/home).
